# Online network organization of Barcelona en Comú, an emergent movement-party

**DOI:** 10.1186/s40649-017-0044-4

**Published:** 2017-09-18

**Authors:** Pablo Aragón, Helena Gallego, David Laniado, Yana Volkovich, Andreas Kaltenbrunner

**Affiliations:** 1 Universitat Pompeu Fabra, Barcelona, Spain; 2 Eurecat-Technology Centre of Catalonia, Avinguda Diagonal, 177, 08018 Barcelona, Spain

**Keywords:** Twitter, Politics, Social movements, Political parties, 15M movement, Indignados movement, Spanish elections, Online campaigning

## Abstract

The emerging grassroots party Barcelona en Comú won the 2015 Barcelona City Council election. This candidacy was devised by activists involved in the Spanish 15M movement to transform citizen outrage into political change. On the one hand, the 15M movement was based on a decentralized structure. On the other hand, political science literature postulates that parties develop oligarchical leadership structures. This tension motivates to examine whether Barcelona en Comú preserved a decentralized structure or adopted a conventional centralized organization. In this study we develop a computational methodology to characterize the online network organization of every party in the election campaign on Twitter. Results on the network of retweets reveal that, while traditional parties are organized in a single cluster, for Barcelona en Comú two well-defined groups co-exist: a centralized cluster led by the candidate and party accounts, and a decentralized cluster with the movement activists. Furthermore, results on the network of replies also shows a dual structure: a cluster around the candidate receiving the largest attention from other parties, and another with the movement activists exhibiting a higher predisposition to dialogue with other parties.

## Background

The last decade has seen a global wave of citizen protests: the Arab Spring, the 15M movement in Spain, Occupy Wall Street, #YoSoy132 in Mexico, Occupy Gezi in Turkey, the Brazilian movement #VemPraRua, Occupy Central in Hong Kong, etc. All these movements share common characteristics such as the claim for new models of democracy, the strategic usage of social media (e.g., Twitter), and the occupation of physical public spaces. One of the weaknesses of these movements is their difficulty in accessing institutions and impacting public policies. The 2015 Barcelona City Council election is one of the first cases in which one of these movements has been able to “occupy” the public institutions by building Barcelona en Comú (BeC), a political party that won the elections. BeC was conceived as the confluence of (1) minor and/or emerging parties and, to a large extent, (2) collectives and activists, with no political party affiliation, who played a prominent role in the 15M movement.

The 15M movement, also referred to as #SpanishRevolution or the “Indignados” movement, emerged in May 2011 and has been defined as a “networked social movement of the digital age” [13]. Networked social movements, like the Arab Spring, the 15M, and Occupy Wall Street, are claimed to be “a network of networks, they can afford not to have an identifiable center, and yet ensure coordination functions, as well as deliberation, by interaction between multiple nodes” [13]. Other authors have defined this new model of social movement as a “change from logic of collective action, associated with high levels of organizational resources and the formation of collective identities, to a logic of connective action, based on personalized content sharing across media networks” [7]. There, these can be seen as paradigmatic examples of how the Internet is able to alter the mobilizing structure for collective action [50]. We should note that some voices have refused these theoretical assumptions and argued that “a handful of people control most of the communication flow” and, consequently, the existence of leaders in such movements could not be denied [27]. Empirical studies revealed that the 15M network on Twitter is characterized by its “decentralized structure, based on coalitions of smaller organizations” in spite of “a small core of central users is still critical to trigger chains of messages of high orders of magnitude” [30]. Decentralization has been also observed in [59] in which the 15M network is defined as polycentric.

The 15M network properties (i.e., decentralization, polycentrism) could be perceived as a striking contrast to conventional political organizations, in particular, political parties. The Iron Law of Oligarchy [43] postulates that political parties, like any complex organization, self-generate an elite (i.e., “Who says organization, says oligarchy”). Although some scholars have criticized the idea that organizations will intrinsically build oligarchical leadership structures [18, 37, 55], many political and social theorists have supported that, historically, small minorities hold the most power in political processes [44, 46, 51]. At the interplay between politics and the Internet, different studies have found the frequent presence of elites [19, 57]. Regarding Spanish online politics, a study of the 2011 national election campaign on Twitter revealed that “minor and new parties tend to be more clustered and better connected, which implies a more cohesive community” [5]. Nevertheless, all the diffusion networks of parties in that study were strongly centralized around their candidate and/or party profiles. Later studies analyzed the interactions on Twitter between the 15M nodes and political parties and conclude that networked social movements are *para-institutions*: perceived as institutions but preserving an internal networked organization [52]. However, these conclusions were formulated by analyzing the networks when no elections were held, before institutionalization began. Election campaigns are competitive processes that might favor the centralization of an organization around candidates. Indeed, it has been proved that the network properties of political parties change when elections arrive [23]. Previous hypotheses [58] about Podemos, a member party of the Barcelona en Comú candidacy and as well inspired by the 15M movement, postulate an organization formed by a *front-end* (“spokesmen/spokeswomen who are visible from the media perspective”) and a *back-end* (“muscle of the organization, barely visible from the media perspective”). However, there are no empirical validations of this hypothesis.

Given that Barcelona en Comú emerged from the 15M and this networked movement is characterized by a decentralized structure, the first research question of this study is:

RQ1: Has Barcelona en Comú preserved a decentralized structure or has it adopted a conventional centralized organization ruled by an elite?

To answer our first research question, we will analyze the network of retweets in relation to the campaign for the 2015 Barcelona City Council election to (1) identify clusters of political parties and (2) characterize their topology. The identification of the sub-network corresponding to each party will be possible because of the highly divided partisan structure of the retweet network. This assumption relies on previous studies of online polarization in social media in the context of US politics [1, 16]. Online polarization, also known as cyberbalkanization, is a social phenomenon that occurs when Internet users form isolated groups around specific, e.g., political interests. Indeed, this is not only a particular feature of US politics but also a social behavior observed in a diverse range of countries, e.g., Canada [31] and Germany [20]. In Spain, previous studies of Twitter networks in previous elections also showed evidence of polarization, e.g., in the 2010 Catalan election [15] and in the 2011 Spanish elections [11].

We also find of interest to explore the behavior of Barcelona en Comú when discussing with other political parties. The 15M movement, which motivated the emergence of this grassroots party, is characterized by its willingness to expand the practices of deliberative democracy beyond institutions [54]. Indeed, recent studies about the internal communication of Barcelona en Comú have already shown the relevance of discussions in online platforms [10, 32]. In contrast, previous research found little dialogue between the 15M movement and political institutions, with sporadic exceptions with minor and left-wing parties [52]. Given that Barcelona en Comú, as any political party, is expected to discuss with other political parties, the second research question of the study is:

RQ2: Does Barcelona en Comú discuss differently with other political parties than traditional parties do?

The extent of political polarization that can be observed in social media depends on the kind of interaction considered. In the case of the American political blogosphere, a seminal work by Adamic et al. [1] showed that few links connected liberal and conservative blogs, as bloggers mostly refer to ideologically related others. On the contrary in Wikipedia, a platform where users editing the same articles are brought to discuss and pursue consensus, partisan users were observed to be equally likely to interact with others supporting the same or the opposite party [48]. Likewise, in the case of Twitter different results have been observed for retweets and reply networks. Retweeting has been proven as a common mechanism for endorsement [12] which might explain why retweet networks exhibit polarization to a greater extent than reply networks [5, 16]. This is consistent with the results from a study of a Swiss political online platform which concluded that “interactions with positive connotation (supports and likes) revealed significant patterns of polarization with respect to party alignment, unlike the comments layer, which has negligible polarization” [23]. To answer this second question, we will therefore examine the online party discussion networks by analyzing the network of replies and comparing the structure of clusters to the ones from the network of retweets.

This article is organized as follows. In “Methods” section we describe the techniques of our methodology to detect clusters in Twitter networks and to characterize their topology. The dataset of tweets related to the 2015 Barcelona City Council election is described in “Dataset” section. We present in “Online party organization networks” section the results of our methodology using the network of retweets. A similar analysis on the network of replies is shown in “Online party discussion networks” section. In “Discussion” section we discuss the results of the analysis to answer our research questions about the online structure of Barcelona en Comú and the interaction of this new organization towards traditional parties. Finally, we conclude in “Conclusion” section.

## Methods

Here we describe the methodology of our study to, given a network, detect the major clusters (i.e., political parties) and characterize their social structures in three dimensions: hierarchical structure, small-world phenomenon, and coreness.

### Community detection

Many previous studies have relied on the Louvain method [9] because of its high performance in terms of accuracy, and its efficiency. This method is based on a greedy algorithm that attempts to optimize the modularity of a partition of a given network. Modularity function measures the density of edges inside communities in comparison to edges between communities [49]. Given a network, the modularity value, lying between −1 and 1, is defined as:

$$\begin{aligned} Q = \frac{1}{2m}\Sigma _{ij}\bigg [A_{ij} - \frac{d_i d_j}{2m}\bigg ]\delta (c_i,c_j), \end{aligned}$$

where $A_{ij}$ is the edge weight between nodes *i* and *j*; $d_i$ and $d_j$ are the degrees of the nodes *i* and *j*, respectively; *m* represents the total number of edges in the network; $c_i$ and $c_j$ are the indexes of communities of those nodes; and $\delta$ is the Kronecker delta.

The Louvain method follows a two-step approach. First, each node is assigned to its own community. Then, for each node *i*, the change in modularity is measured for moving *i* from its own community into the community of each neighbor *j*:

$$\begin{aligned} \Delta Q = \bigg [ \frac{S_\mathrm{in} + w_{i,\mathrm in}}{2m} - \bigg (\frac{S_\mathrm{tot} + d_i}{2m}\bigg )^2 \bigg ]-\bigg [\frac{S_\mathrm{in}}{2m} - \bigg (\frac{S_\mathrm{tot}}{2m}\bigg )^2-\bigg (\frac{d_i}{2m}\bigg )^2\bigg ], \end{aligned}$$

where $S_\mathrm{in}$ is the sum of all the weights of the intra-edges of the community where *i* being moved into, $S_\mathrm{tot}$ is the sum of all the weights of the edges to nodes of the community, $d_i$ is the degree of *i*, $w_{i,{\rm in}}$ is the sum of the weights of the edges between *i* and other nodes in the community, and *m* is the sum of the weights of all edges in the network. Once this value is measured for all communities that *i* is linked to, the algorithm sets *i* into the community that produces the largest increase in modularity. If no increase is possible, *i* remains in its original community. This process is applied until modularity cannot be increased and a local maximum of modularity is achieved. Then, the method groups the nodes from the same community and builds a new network where nodes are the communities from the previous step. Both steps are repeated until modularity cannot be increased.

#### N-Louvain method

The Louvain method is a greedy algorithm and has a random component, so each execution produces a different result. To obtain robust results, avoiding dependency on a particular execution of the algorithm, this article introduces the following modification to identify the main clusters of the network in a robust way.

First, it runs *N* executions of the Louvain algorithm, which produce *N* different partitions of the network into clusters. To identify each cluster across executions, our method applies the Jaccard index [33] to every pair of clusters $c_i$ and $c_j$ from different executions:

$$\begin{aligned} J(c_i,c_j) = { |c_i \cap c_j| \over |c_i \cup c_j| }. \end{aligned}$$

Thus, clusters across executions are matched if they are the most similar ones. This allows to quantify the occurrences (i.e., executions) of each node in each cluster. Finally, the method assigns to each cluster all the nodes that appear in that cluster in at least a fraction $(1-\varepsilon )$ of the partitions created, that is to say that $\varepsilon$ represents the sensibility level of the algorithm. This procedure allows to validate the results of the community detection algorithm, and to guarantee that all the nodes that are assigned to a cluster do actually belong to it with a given confidence. The remaining nodes, that cannot be assigned in a stable way to any of the main clusters, are left out from all the clusters.

### Cluster characterization

Inspired by the social dimensions and corresponding metrics suggested in [23] we propose an extended framework to compare the topology of the intra-network of each cluster.

#### Hierarchical structure

The hierarchical structure is quantified on the in-degree distribution of each cluster. The in-degree of node *i* is the total number of edges onto node *i*. By counting how many nodes have each in-degree value, the in-degree distribution $P(d_\mathrm{in})$ is equal to the fraction of nodes in the graph with such in-degree $d_\mathrm{in}$. The cumulative in-degree distribution $P(x \ge d_\mathrm{in})$ represents the fraction of nodes in the graph whose in-degree is greater than or equal to $d_\mathrm{in}$.

The original framework [23] used an existing method to measure degree centralization defined in [22]. Degree centralization is based on two concepts:

1. How the centrality of the most central node exceeds the centrality of all other nodes.
2. Setting the value as a ratio by comparing to a star network:
  $$\begin{aligned} C_\mathrm{in} = \frac{\sum \nolimits _{i=1}^n [d^\mathrm{in}_{\max } - d^\mathrm{in}_{i}]}{ \max \sum \nolimits _{i=1}^n [d^\mathrm{in}_{\max } - d^\mathrm{in}_{i}] } , \end{aligned}$$
  where $d^\mathrm{in}_{i}$ is the in-degree of node *i*, $d^\mathrm{in}_{\max }$ is the maximum in-degree of the network, and $\max \sum \nolimits _{i=1}^n [d^\mathrm{in}_{\max } - d^\mathrm{in}_{i}]$ is the maximum possible sum of differences for a graph with the same number of nodes (a star network).

The differences of several orders of magnitude between the maximum and average in-degree, which characterize social graphs, make this metric approximately equal to the ratio between the maximum in-degree and the number of nodes:

$$\begin{aligned} C_\mathrm{in} = \frac{\sum \nolimits _{i=1}^n [d^\mathrm{in}_{\max } - d^\mathrm{in}_{i}]}{ \max \sum \nolimits _{i=1}^n [d^\mathrm{in}_{\max } - d^\mathrm{in}_{i}]} \approx \frac{(n-1) \cdot d^\mathrm{in}_{\max }}{(n-1) \cdot (n-1)} \approx \frac{d^\mathrm{in}_{\max }}{n} \end{aligned}.$$

Therefore, to better evaluate the hierarchical structure of graphs, we will also apply the Gini coefficient, a statistical metric to quantify the level of inequality given a distribution [28]. It was initially formulated in Economics to measure the income distribution using the Lorenz curve. The Gini coefficient is equal to

$$\begin{aligned} G_\mathrm{in}=A/(A+B), \end{aligned}$$

where *A* is the area between the line corresponding perfect equality and *B* is the area under the Lorenz curve. If the Lorenz curve is expressed by the function $y = L(x)$, *B* is calculated as $B = 1 -2 \int _0^1 L(x)\, {\mathrm{d}}x$ and $A=1/2-B$. In the context of network topology, the Gini coefficient is applied to characterize the hierarchical structure of a network based on the inequality of its in-degree distribution.

#### Small-world phenomenon

The small-world phenomenon states that most nodes of a network are reachable from any other node in a small number of steps and explains information efficiency in social networks. To assess the small-world phenomenon in each cluster, the clustering coefficient and the average path length are computed. Small-world networks tend to have a small average path length and a clustering coefficient significantly higher than expected by random chance [62]. The clustering coefficient measures the extent of nodes to cluster together by calculating the number of triangles in the network. For every node *i* it sets $N_i$ to be the neighborhood, i.e., $N_i=\{j\in V: (i,j)\in E\}$, and defines the local clustering coefficient as

$$\begin{aligned} \mathrm{Cl}_i=\frac{2|(j,k)\in E: j,\;k\in N_i|}{k_i(k_i-1)}. \end{aligned}$$

Then, following [62], the clustering coefficient is just the average of the local clustering coefficients: $\mathrm{Cl}=\sum _{i}\mathrm{Cl}_i/n,$ where *n* is the number of nodes in the network. To calculate the average path length, for every pair of nodes *i* and *j*, it sets $\ell _{ij}$ to be the smallest number of steps among all paths between *i* and *j*. This metric is applied to the clusters identified by the new version algorithm for community detection and, by definition, there is always a path between any pair of nodes in every cluster. The average path length is defined as follows:

$$\begin{aligned} l={\sum _{i\ne j}\ell _{ij}}/{n(n-1)} \end{aligned}.$$

#### Coreness

Coreness has been employed in previous literature as a metric of the resilience of a network [24]. The resilience of a social network is the ability of a social group to withstand external stresses. To measure coreness of the intra-network of each cluster the *k*-core decomposition is applied in order to evaluate the distributions of the nodes within each *k*-core.

Given a network, a sub-network *H* induced by the subset of nodes *C* is defined. *H* is a *k*-core of the network if and only if for every node *i* in *C*: $\mathrm{deg}_H(i) \ge k,$ and *H* is the maximum sub-graph which fulfills this condition. The degree of the node *i* in the sub-graph *H* is denoted as $\mathrm{deg}_H(i)$. A node has *k*-index equal to *k* if it belongs to the *k*-core but not to the $(k+1)$-core. In simple words, *k*-core decomposition starts with $k = 1$ and removes all nodes with degree equal to 1. The procedure is repeated iteratively until no nodes with degree 1 remain. Next, all removed nodes are assigned *k*-index to be 1. It continues with the same procedure for $k = 2$ and obtains nodes with indexes equal 2, and so on. The process stops when the last node from the network is removed at the ${k_\mathrm{max}}\,{\rm th}$ step. The variable $k_\mathrm{max}$ is then the maximum shell index of the graph.

## Dataset

Data were collected from Twitter in relation to the campaign for the 2015 Barcelona City Council election (May 1–26, 2015) by the definition of a list of Twitter accounts of the seven main political parties:

- Barcelona en Comú (BeC),^1^
- Convergència i Unió (CiU),^2^
- Ciudadanos (Cs),^3^
- Capgirem Barcelona (CUP),^4^
- Esquerra Republicana de Catalunya (ERC),^5^
- Partit Popular de Catalunya (PP),^6^
- Partit dels Socialistes de Catalunya (PSC).^7^

The lists also include the Twitter accounts of the corresponding candidates for Mayor. For the case of coalitions (CiU, BeC, and CUP) also the party accounts of the parties constituting the coalition were included. The users of the list can be found in Table 1.

**Table 1:** Twitter accounts of the selected political parties and candidates

Political party	Party account(s)	Candidate account
	@bcnencomu	
	@icveuiabcn	
BeC	@podem_bcn	@adacolau
	@equobcn	
	@pconstituentbcn	
CiU	@cdcbarcelona	@xaviertrias
	@uniobcn	
Cs	@cs_bcna	@carinamejias
CUP	@capgirembcn	@mjlecha
	@cupbarcelona	
ERC	@ercbcn	@alfredbosch
PP	@ppbarcelona_	@albertofdezxbcn
PSC	@pscbarcelona	@jaumecollboni

It is important to note that the sampling criteria are based on specific accounts instead of hashtags. Some studies have detected differences in the tagging practice of politicians [36]. Previous work has observed that some parties adopt a small set of hashtags during campaigns and some other parties generate new hashtags every day in order to locate them in the list of trending topics. Therefore, sampling messages from a list of campaign hashtags would likely lead to an unbalanced dataset. For this reason, we believe these sampling criteria represent a better approach to capture the communication practices of the communities around parties.

**Fig. 1:**
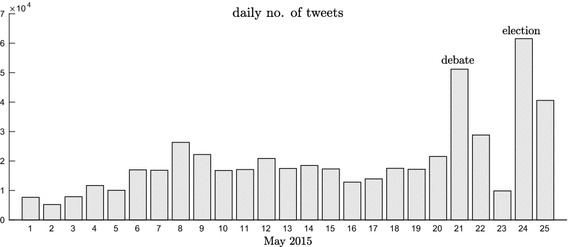
Distribution of the number of tweets in the dataset over time

The Twitter streaming API provided 507,597 tweets that (1) were created by, (2) retweeted, or (3) mentioned an account from the list. Figure 1 shows the distribution of the tweets over time, and reveals that the most active dates were the election day (March 24) and the one of the televised debate between candidates (March 21). In contrast, the day preceding the election, known as the reflection day, shows a notable decrease in Twitter activity. This distribution is similar to the one observed in previous studies about Spanish politics on this social network [5].

To detect and characterize the online network organization of political parties, we build a directed weighted graph which comprises a set of nodes (users) and a set of edges (retweets between any pair of users). Each edge in the graph represents that the source user retweeted a message posted by the target user. To exclude anecdotal interactions between users which might not be enough of a signal to infer endorsement [25] and to highlight the structure of the expected clusters, the network only contains the interactions between any pair of nodes that occurred at least 3 times: an edge from user A to user B implies that user A has retweeted at least 3 times user B in the dataset. We considered a threshold of 3 retweets to be strong enough for inferring endorsement, and for filtering out anecdotal interactions without loosing relevant connections. Nodes without edges after this process are removed. The resulting retweet network comprises 6492 nodes and 16,775 edges.

To analyze the discussions between political parties, we built another directed weighted graph, being in this case the edges of the graph replies instead of retweets. Unlike the retweet graph, where the interactions have been filtered by 3, the reply graph is maintaining all the edges. The motivation for this lies in the different nature of replies: while a single retweet could be anecdotal and has a low cost for a user (one click), a reply is a more expensive action involving more cognitive overhead, which makes it a noteworthy interaction already if it happens only once [47]. Indeed, previous work has found that retweeting has a higher likelihood than replying to a tweet [3]. The resulting reply network consists of 21,846 nodes and 44,598 edges.

## Online party organization networks

In this section we present the results of detecting and characterizing the major clusters in the network of retweets, i.e., the online party organization networks.

### Community detection

To detect the online organization network of each political party, we apply the N-Louvain method. This new version has been designed to detect clusters which only include nodes that are reliably assigned to them. We apply the method by running the Louvain method 100 times and assigning to each cluster only the nodes that fall into that cluster more than 95 times ($N=100, \varepsilon =0.05$). By inspecting the results of the 100 executions, a constant presence of eight major clusters, much bigger than the other clusters, is observed. The composition of these clusters is also quite stable: 4973 nodes (82.25%) are assigned to the same cluster in over 95 executions.

We examine the most relevant nodes of every cluster, according to PageRank, and find a single cluster for almost each party: ${\rm ERC ^{\rm{rt}}}$, ${\rm CUP ^{\rm{rt}}}$, ${\rm Cs ^{\rm{rt}}}$, ${\rm CiU ^{\rm{rt}}}$, ${\rm PP ^{\rm{rt}}}$, and ${\rm PSC ^{\rm{rt}}}$. The only exception for such rule is that BeC is composed of two clusters. The manual inspection of the users from these two clusters reveals that one cluster is formed by the official accounts of the party (e.g., @bcnencomu, @ahorapodemos), allied parties (e.g., @ahoramadrid), the candidate (@adacolau), and a large community of peripheral users. In contrast, the other cluster is composed of activists engaged in the digital communication for the campaign (e.g., @toret, @santidemajo, @galapita), i.e., party activists, many of whom are related to the 15M movement. For this reason, from now on, the analysis distinguishes these clusters as ${\rm BeC{\text{-}}p ^{\rm{rt}}}$ and ${\rm BeC{\text{-}}m ^{\rm{rt}}}$: *party* and *movement*, respectively.

Table 2 shows the top five users with highest PageRank in each cluster, and their role with respect to the corresponding party: *candidate* (the account of the candidate for mayor), *party* (official accounts of parties associated with the candidacy), *activist* (party activists), *institution* (institutional accounts), *media* (accounts of media or journalists). It should be noted that we also considered the category *politician* to distinguish professional politicians from activists; however, no politician with an institutional position was found among the top five users from each cluster. While the topmost relevant users tend to correspond to each party’s candidate and official accounts, which is partly caused by the data collection criteria, it is interesting to note the presence of other very central nodes in these clusters, including media or institutional accounts (the municipality account, in the cluster of the outgoing mayor’s party). BeC-m is the only cluster for which the top users are mostly activists.

**Table 2:** Top 5 users for the 8 largest clusters according to their PageRank in the overall network, with their role with respect to the corresponding party

Cluster	User	PageRank	Role
BeC-p	@bcnencomu	0.092	Party
BeC-p	@adacolau	0.029	Candidate
BeC-p	@ahoramadrid	0.009	Allied party
BeC-p	@ahorapodemos	0.009	Party
BeC-p	@isaranjuez	0.002	Activist
BeC-m	@toret	0.014	Activist
BeC-m	@santidemajo	0.005	Activist
BeC-m	@sentitcritic	0.005	Media
BeC-m	@galapita	0.005	Activist
BeC-m	@eloibadia	0.005	Activist
Cs	@carinamejias	0.007	Candidate
Cs	@cs_bcna	0.006	Party
Cs	@ciudadanoscs	0.004	Party
Cs	@soniasi02	0.003	Activist
Cs	@prensacs	0.002	Party
CiU	@xaviertrias	0.012	Candidate
CiU	@ciu	0.004	Party
CiU	@bcn_ajuntament	0.003	Institution
CiU	@cdcbarcelona	0.002	Party
CiU	@uniobcn	0.001	Party
CUP	@cupbarcelona	0.016	Party
CUP	@capgirembcn	0.008	Party
CUP	@albertmartnez	0.005	Media
CUP	@mjlecha	0.002	Candidate
CUP	@simongorjeos	0.003	Media
ERC	@ercbcn	0.016	Party
ERC	@alfredbosch	0.011	Candidate
ERC	@arapolitica	0.007	Media
ERC	@esquerra_erc	0.004	Party
ERC	@directe	0.003	Media
PP	@cati_bcn	0.003	Media
PP	@albertofdezxbcn	0.003	Candidate
PP	@maticatradio	0.002	Media
PP	@ppbarcelona_	0.002	Party
PP	@carmenchusalas	0.001	Activist
PSC	@pscbarcelona	0.003	Party
PSC	@sergifor	0.003	Media
PSC	@jaumecollboni	0.002	Candidate
PSC	@elpaiscat	0.002	Media
PSC	@annatorrasfont	0.001	Media

**Fig. 2:**
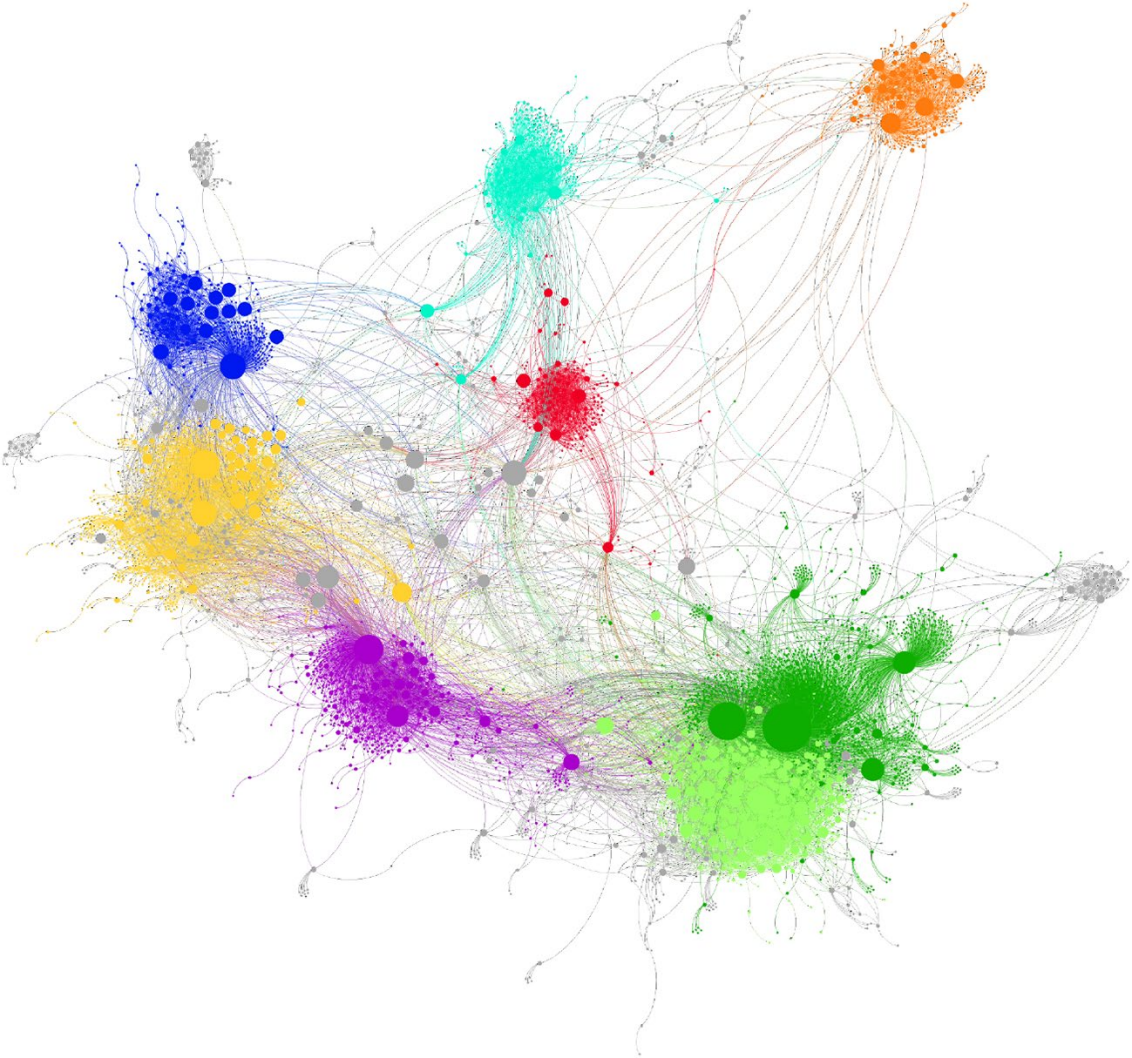
Network of retweets (giant component). Clusters are represented by color: ${\rm BeC \text{-}p ^{\rm{rt}}}$ (*dark green*); ${\rm BeC \text{-}m ^{\rm{rt}}}$ (*light green*); ${\rm ERC ^{\rm{rt}}}$ (*yellow*); ${\rm PSC ^{\rm{rt}}}$ (*red*); ${\rm CUP ^{\rm{rt}}}$ (*violet*); ${\rm Cs ^{\rm{rt}}}$ (*orange*); ${\rm CiU ^{\rm{rt}}}$ (*dark blue*); ${\rm PP ^{\rm{rt}}}$ (*cyan*). The nodes outside of these clusters are *gray* colored

**Fig. 3:**
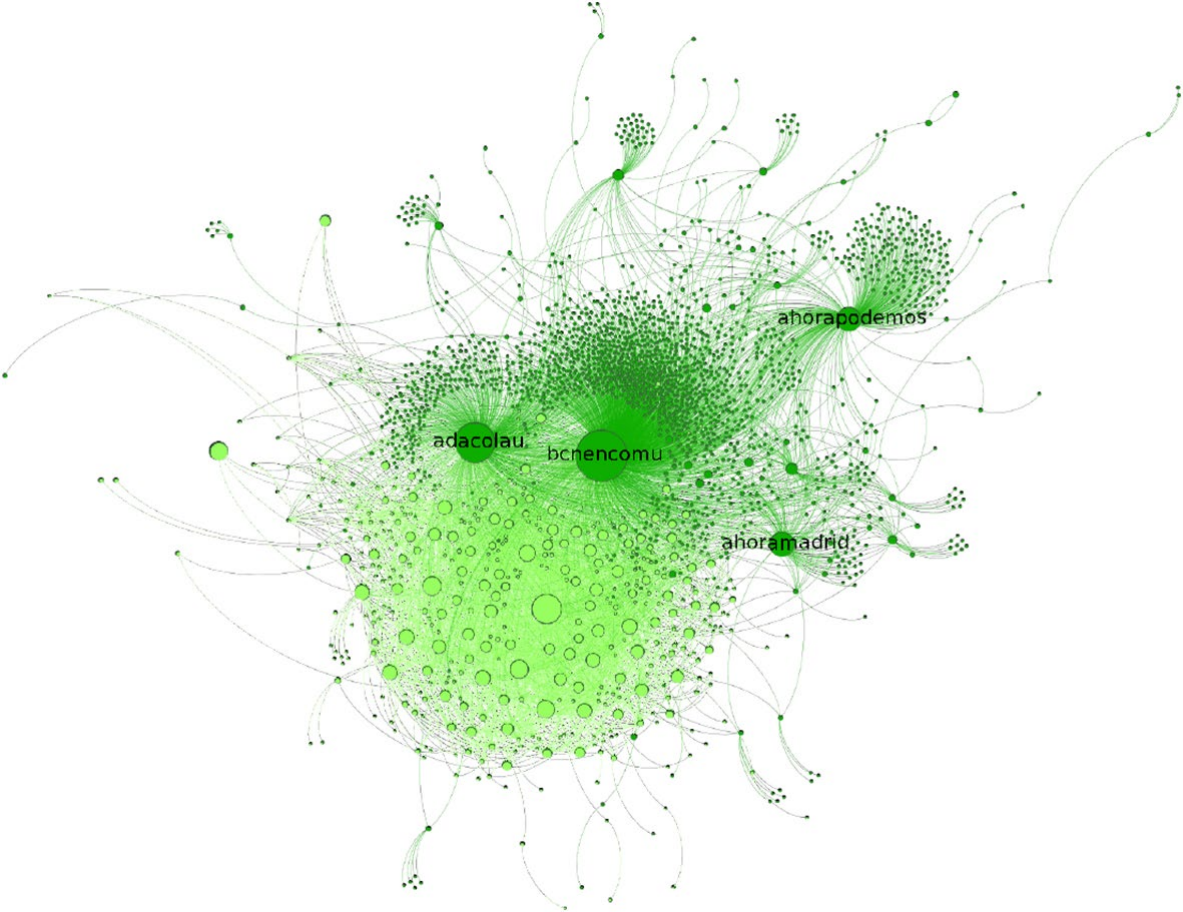
Sub-network of ${\rm BeC \text{-}p ^{\rm{rt}}}$ (*dark green*) and ${\rm BeC \text{-}m ^{\rm{rt}}}$ (*light green*). For better readability, the labels of public figures are shown

**Fig. 4:**
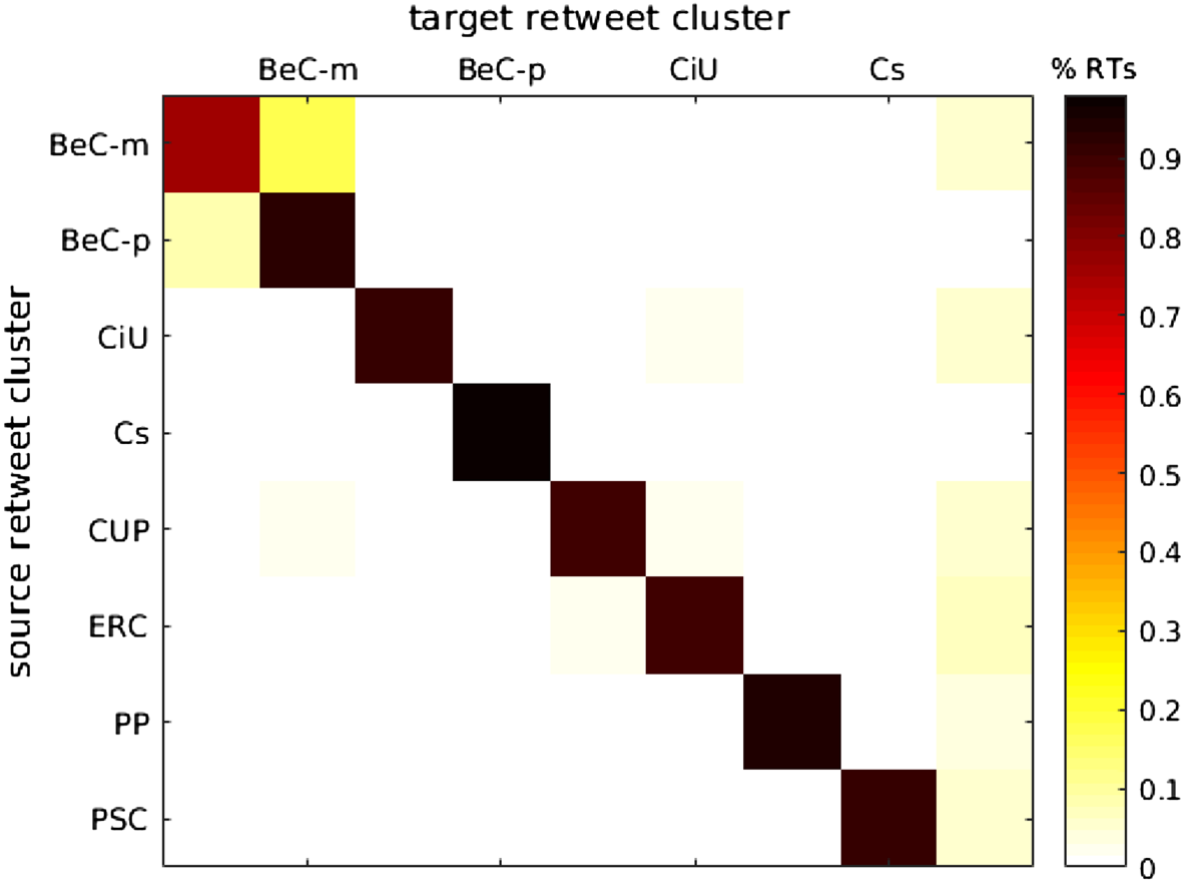
Normalized weighted adjacency matrix of the network retweets grouping nodes by clusters

The boundaries between ideological online communities are visible in Fig. 2. As one could expect in any polarized scenario, the largest number of retweets occur within the same cluster. There exists, however, a notably large number of links between the two clusters of BeC (${\rm BeC \text{-}p ^{\rm{rt}}}$, and ${\rm BeC\text{-}m ^{\rm{rt}}}$). Figure 3 presents the sub-network formed by the nodes and links of both clusters. To further prove the low levels of interactions between major parties, an interaction matrix *A* is defined, where $A_{i,j}$ counts all retweets that accounts from cluster $i^\mathrm{rt}$ made for the tweets from users of cluster $j^\mathrm{rt}$. Since the clusters have different sizes, $A_{i,j}$ is normalized by the sum of the all retweets made by the users assigned to cluster *i*. Figure 4 shows matrix *A* for all the clusters and confirms that a vast majority of retweets were made between users from the same cluster (main diagonal). This is also true in the case of the two clusters of Barcelona en Comú although there is a presence of communication between movement and party clusters, with a prevalence from the movement to the party $({\rm BeC \text{-}m ^{\rm{rt}}} \rightarrow {\rm BeC \text {-}p ^{\rm{rt}}} = 0.18)$, the largest value out of the main diagonal.

As mentioned above, the new version of the Louvain method proposed in this article only assigns a node to one of the eight largest clusters only if it falls to a particular one of these clusters more than 95 of 100 times. The final inclusion/exclusion of the most relevant nodes to a cluster was manually inspected in order to assess the performance of this new version. For preserving the political preference of non-public users, Table 3 only presents the 20 most relevant nodes which were not assigned to any cluster, their role, and how many times they fall into each cluster over the 100 executions. The results prove that N-Louvain method effectively prevented the inclusion of media accounts in the intra-network of political parties, e.g., @btvnoticies, @elperiodico, @elsmatins, etc. Also, for better readability, when a node falls in different political clusters more than 20% each, we highlight the corresponding values in Table 3. First, we observe that the Catalan pro-independence media outlet @naciodigital and two journalists from that outlet (@bernatff, @jordi_palmer) fell in ${\rm ERC ^{\rm{rt}}}$ and ${\rm CUP ^{\rm{rt}}}$, i.e., clusters of Catalan pro-independence parties. Second, we find that the TV show @puntcattv3 fell in ${\rm ERC ^{\rm{rt}}}$ and ${\rm PSC ^{\rm{rt}}}$ and the media outlet @xriusenoticies in ${\rm CiU ^{\rm{rt}}}$ and ${\rm PSC ^{\rm{rt}}}$. Results also show that @mariamariekke, a citizen who created drawings for the BeC campaign, fell between the two clusters of the party (party and movement). Finally, we also find of interest the appearance of civic organizations: *Plataforma de Afectados por la Hipoteca* mostly in ${\rm BeC \text{-}m ^{\rm{rt}}}$ (organization to stop evictions which was co-founded by the candidate of BeC), and Vaga de Totes (feminist labor organization), which lies between the left parties ${\rm BeC \text{-}m ^{\rm{rt}}}$ and ${\rm CUP ^{\rm{rt}}}$.

**Table 3:** Most relevant nodes, according to PageRank, which could not be reliably assigned to any of the major clusters indicating the number of executions in each cluster

User	Role	${\rm BeC \text{-}m ^{\rm{rt}}}$	${\rm BeC \text{-}p ^{\rm{rt}}}$	${\rm CiU ^{\rm{rt}}}$	${\rm Cs ^{\rm{rt}}}$	${\rm CUP ^{\rm{rt}}}$	${\rm ERC ^{\rm{rt}}}$	${\rm PP ^{\rm{rt}}}$	${\rm PSC ^{\rm{rt}}}$	Undef.
@btvnoticies	Media	0	0	0	0	1	0	86	13	0
@elperiodico	Media	0	90	0	3	0	1	0	1	5
@elsmatins	Media	0	0	0	0	0	93	0	7	0
@naciodigital	Media	0	0	1	0	*38*	*61*	0	0	0
@tv3cat	Media	0	0	0	0	3	54	0	19	24
@encampanya	Media	1	0	0	0	36	0	0	0	63
@rocsalafaixa	Citizen	0	0	7	0	1	92	0	0	0
@bernatff	Media	0	0	1	0	*38*	*61*	0	0	0
@jordi_palmer	Media	0	0	1	0	*38*	*61*	0	0	0
@mariamariekke	Citizen	*50*	*50*	0	0	0	0	0	0	0
@puntcattv3	Media	0	0	0	0	0	*44*	0	*56*	0
@ramontremosa	Politician	0	0	90	0	0	10	0	0	0
@santimdx5	Media	91	0	0	0	0	0	0	0	9
@mtudela	Media	0	0	7	0	1	92	0	0	0
@pah_bcn	Civic org	89	0	0	0	0	0	0	0	11
@324cat	Media	0	0	0	0	3	52	0	13	32
@terrassaencomu	Party	2	92	0	0	0	0	0	0	6
@sicomtelevision	Media	1	8	0	0	90	0	0	0	1
@xriusenoticies	Media	0	0	*35*	0	0	0	0	*65*	0
@vagadetotes	Civic org	*78*	0	0	0	*22*	0	0	0	0

Values are italics when a node falls in different political clusters more than 20% each

#### Comparison to the Clique Percolation Method

**Fig. 5:**
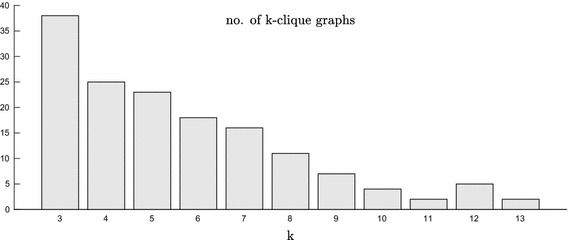
Number of *k*-clique graphs obtained through the Clique Percolation Method for different values of *k*

The design of the N-Louvain method is motivated by the fuzzy community structure of political networks, as one of the campaigns for the 2015 Barcelona City Council election. These networks are usually formed by overlapping communities and the proposed algorithm improves the standard Louvain method by identifying clusters in a more stable way. However, we should note that there are some community detection methods in the state of the art for overlapping communities. In particular, the Clique Percolation Method (CPM) is the most popular one according to [21]. This method is applied on the network of retweets with the CFinder software package^8^ to detect *k*-cliques, i.e., complete (fully connected) sub-graphs of *k* nodes. Figure 5 presents the number of *k*-clique graphs obtained through the CPM at every value of *k*. As expected, the number of *k*-clique graphs tends to decrease as *k* increases. At its maximum value ($k=13$), the method only detects two *k*-clique graphs: one formed by users from BeC and another formed by users from CiU.

While the Louvain method was able to identify every party cluster, CPM at its maximum value only detects two party clusters. This is explained by the different size and structure of the party networks. For this reason, the communities at different values of *k* have been examined. When $k=9$, CPM identifies seven *k*-clique graphs. The inspection of the nodes of each of them reveals that two of them are related to BeC, one is related to a municipal police trade union and the rest are related to each of the political parties CiU, CUP, Cs, and PP. For PSC and ERC, CPM identifies *k*-clique graphs when $k=8$ and $k=7,$ respectively. To compare these results with the clusters from the N-Louvain method, Table 4 indicates how many nodes of the each *k*-clique graph occurred in each cluster, and reveals that:

- All the nodes of the *k*-clique graphs related to CiU, Cs, CUP, ERC, and PSC are part of the corresponding clusters from the N-Louvain method.
- Only one node from PP *k*-clique graph was not in PP political cluster.
- The nodes from the *k*-clique graph related to a trade union of municipal police ($\rm GU$) were not in a political cluster.
- The largest BeC *k*-clique graph ($\rm BeC_1$) is mainly formed by nodes from the BeC movement cluster. The smallest *k*-clique graph ($\rm BeC_2$) is composed of two nodes from the BeC party cluster and seven nodes from the BeC movement cluster.

**Fig. 6:**
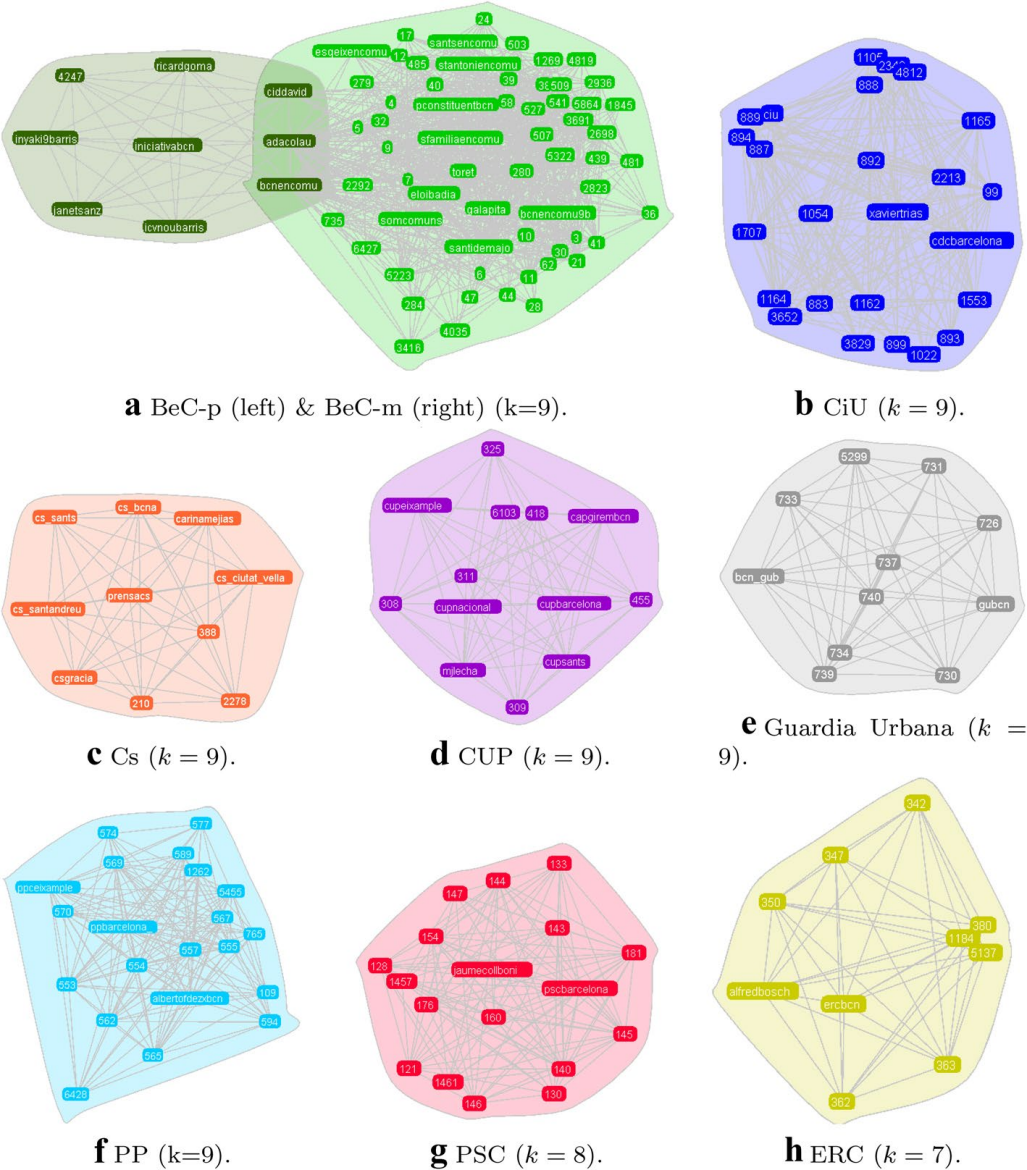
Clique graphs obtained through the Clique Percolation Method. The seven first graphs are the ones when *k* equals to 9. The two last graphs are the largest *k*-clique graphs for PSC ($k=8$), and ERC ($k=7$). Accounts of non-public citizens are anonymized by showing a numerical ID in the label

Figure 6 presents all these *k*-clique graphs to better understand their composition. The figure shows an overlap between the two BeC *k*-clique graphs which is composed of three nodes: @bcnencomu (party account), @adacolau (candidate), and @ciddavid (party member). It is interesting to observe that, although the rest of the nodes of the smallest *k*-clique graph belongs to the movement cluster, all of them are related to Iniciativa per Catalunya Verds, the main pre-existing party that converged in Barcelona en Comú. In other words, CPM also identifies a *k*-clique graph related to the institutional elite of BeC and a much larger *k*-clique graph related to the grassroots elements of BeC.

**Table 4:** Clusters obtained through Clique Percolation Method, *k* value of *k*-clique graph, and number of nodes which occur in the clusters obtained through the N-Louvain method

CPM	*k*	$\text{BeC-m}^{\rm{rt}}$	$\text{BeC-p}^{\rm{rt}}$	$\text{CiU}^{\rm{rt}}$	$\text{Cs}^{\rm{rt}}$	$\text{CUP}^{\rm{rt}}$	$\text{ERC}^{\rm{rt}}$	$\text{PP}^{\rm{rt}}$	$\text{PSC}^{\rm{rt}}$	Undef.
${\rm BeC_{1}}$	9	*60*	3	0	0	0	0	0	0	2
${\rm BeC_{2}}$	9	*7*	2	0	0	0	0	0	0	0
CiU	9	0	0	*25*	0	0	0	0	0	0
Cs	9	0	0	0	*10*	0	0	0	0	0
CUP	9	0	0	0	0	*13*	0	0	0	0
ERC	7	0	0	0	0	0	*7*	0	0	0
PP	9	0	0	0	0	0	0	*20*	0	1
PSC	8	0	0	0	0	0	0	0	*18*	0
GU	9	0	0	0	0	0	0	0	0	*11*

The largest number of each row is italics

In conclusion, the results from applying CPM are consistent with the ones obtained through the community detection algorithm proposed in this article. However, the N-Louvain method has two substantial advantages over CPM:

- The different size and structure of the political networks make that CPM at the maximum value of *k* only detects two major clusters. On the other hand, the new method is able to identify every party cluster.
- The clusters obtained through CPM are *k*-cliques and, therefore, such clusters are dense graphs formed by the core of the party network structure. Social networks are characterized by their heavy-tailed degree distribution so the *k*-clique graphs exclude the large amount of less active users. Recent studies have proved that these are the nodes which compose the critical periphery in the growth of protest movements [6]. For this reason, the inclusion of these nodes, as the new method does, becomes essential for the following characterization of clusters.

### Cluster characterization

The eight clusters detected by the community detection algorithm are then characterized in terms of hierarchical structure, small-world phenomenon, and coreness.

#### Hierarchical structure

To evaluate the hierarchical structure, the in-degree inequality of each cluster is measured with the Gini coefficient. In-degree centralization, originally suggested in [23], is also computed.

**Fig. 7:**
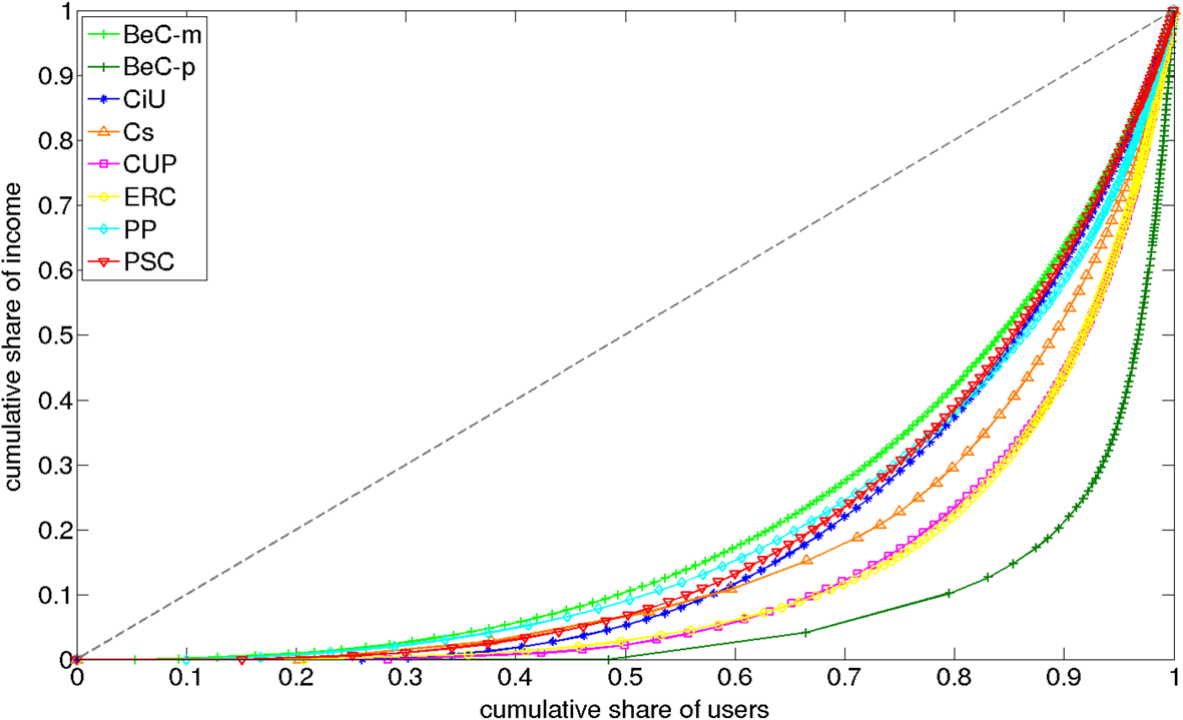
Lorenz curve of the in-degree distribution of each cluster in the network of retweets

From results in Table 5 a notable divergence between both metrics is seen: the inequality values of ${\rm CiU ^{\rm{rt}}}$ and ${\rm PP ^{\rm{rt}}}$ are similar ($G_\mathrm{in}=0.893$ and $G_\mathrm{in}=0.876$, respectively), but the centralization of ${\rm PP ^{\rm{rt}}}$ ($C_\mathrm{in}=0.378$) is far from the maximum centralization value exhibited by ${\rm CiU ^{\rm{rt}}}$ ($C_\mathrm{in}=0.770$). For Barcelona en Comú, ${\rm BeC \text{-}m ^{\rm{rt}}}$ emerges as the least inequal and the least centralized structure, while ${\rm BeC \text{-}p ^{\rm{rt}}}$ forms the most inequal cluster ($G_\mathrm{in}=0.995$). The results in Table 5 confirm that the in-degree centralization formulated in [22] is almost equal to the ratio between the maximum in-degree and the number of nodes. In conclusion, this metric is not a good one to capture hierarchical structure for social diffusion graphs, and the Gini coefficient for in-degree inequality represents a more reliable measure. Finally, the Lorenz curve of the in-degree distribution of the clusters is presented in Fig. 7 to visually validate the different levels of inequality among clusters.

**Table 5:** Inequality based on the Gini coefficient (${ G_{\rm{in}}}$) and centralization (${C_{\rm {in}}}$) of the in-degree distribution of each cluster in the network of retweets, and ratio between the maximum in-degree and the number of nodes (*r*)

Cluster	$G_\mathrm{in}$	$C_\mathrm{in}$	*r*
${\rm BeC \text{-}p ^{\rm{rt}}}$	0.995	0.639	0.639
${\rm Cs ^{\rm{rt}}}$	0.964	0.476	0.480
${\rm ERC ^{\rm{rt}}}$	0.954	0.452	0.454
${\rm CUP ^{\rm{rt}}}$	0.953	0.635	0.636
${\rm CiU ^{\rm{rt}}}$	0.893	0.770	0.774
${\rm PP ^{\rm{rt}}}$	0.876	0.378	0.389
${\rm PSC ^{\rm{rt}}}$	0.818	0.565	0.578
${\rm BeC \text{-}m ^{\rm{rt}}}$	0.811	0.290	0.302

#### Small-world phenomenon

Broadly speaking, the efficiency of a social network is explained by its small-world phenomenon, i.e., phenomenon of users being linked by a mutual acquaintance. To assess the small-world phenomenon in each party, the average path length and the clustering coefficient are computed.

Table 6 reveals that ${\rm BeC \text{-}m ^{\rm{rt}}}$ has the highest clustering coefficient ($\mathrm{Cl}=0.208$) closely followed by ${\rm PP ^{\rm{rt}}}$ and ${\rm PSC ^{\rm{rt}}}$, the two smallest clusters by size. On the contrary the clustering coefficient of ${\rm BeC \text{-}p ^{\rm{rt}}}$ is almost 0. This finding is explained by the topology of ${\rm BeC \text{-}p ^{\rm{rt}}}$, roughly formed by stars whose center nodes are the most visible Twitter accounts of Barcelona en Comú: the party accounts and the candidate.

No remarkable patterns regarding the average path length are observed. It is lower than 3 for the majority of the party clusters with the ${\rm PSC ^{\rm{rt}}}$ cluster having the lowest value ($l=2.29$). At the same time ${\rm ERC ^{\rm{rt}}}$, ${\rm CiU ^{\rm{rt}}},$ and ${\rm BeC \text{-}p ^{\rm{rt}}}$ expose the longest average path length (5.43, 4.66, 3.35, respectively) that might signal the lower information especially in the case of ${\rm ERC ^{\rm{rt}}}$.

**Table 6:** Number of nodes (*N*) and edges (*E*), clustering coefficient (Cl), and average path length (*l*) of the intra-network of each cluster in the network of retweets

Cluster	*N*	*E*	Cl	*l*
${\rm BeC \text{-}m ^{\rm{rt}}}$	427	2431	0.208	3.35
${\rm PP ^{\rm{rt}}}$	301	1163	0.188	2.73
${\rm PSC ^{\rm{rt}}}$	211	810	0.182	2.29
${\rm CiU ^{\rm{rt}}}$	337	1003	0.114	4.66
${\rm Cs ^{\rm{rt}}}$	352	832	0.073	2.57
${\rm CUP ^{\rm{rt}}}$	635	1422	0.037	2.57
${\rm ERC ^{\rm{rt}}}$	866	1899	0.027	5.43
${\rm BeC \text{-}p ^{\rm{rt}}}$	1844	2427	0.002	2.48

#### Coreness

The coreness of a network is closely related to its social resilience, i.e., the ability of a social group to withstand external stresses [23]. To measure social resilience for a social network, the *k*-core decomposition of each cluster is performed in order to evaluate the distributions of the nodes within each *k*-core. The more nodes are in the most inner cores, i.e., the ones with the larger *k*-indexes, and the larger is the maximal *k*-index, then the more resilient the cluster is.

**Fig. 8:**
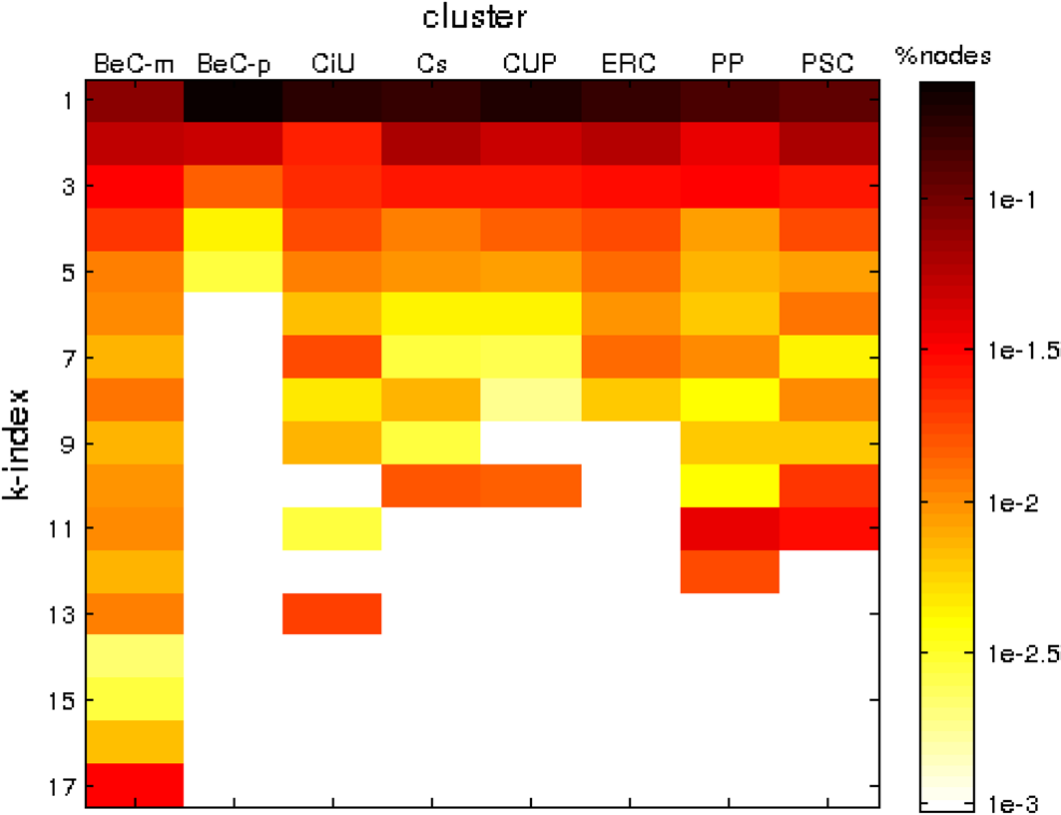
Distribution of the nodes per cluster (*column*) and *k*-index (*row*) in the network of retweets. Cells are colored to form a heat map indicating the percentage of nodes (log scale) from each cluster with a given *k*-index

Table 7 presents the maximal and average *k*-indexes for each cluster and Fig. 8 visually shows the corresponding distributions. As in the case of hierarchical structure and small-world phenomenon, ${\rm BeC \text{-}m ^{\rm{rt}}}$ ($k_\mathrm{max}=17$, $k_\mathrm{avg}=5.90$) and ${\rm BeC \text{-}p ^{\rm{rt}}}$ ($k_\mathrm{max}=5$, $k_\mathrm{avg}=1.33$) are the highest and lowest values, respectively. In comparison to the other parties there are clear differences between node distributions for both, ${\rm BeC \text{-}m ^{\rm{rt}}}$ and ${\rm BeC \text{-}p ^{\rm{rt}}}$, and the rest (the largest concentration of the nodes is in the first *k*-cores and considerable part is in the most inner cores). Therefore, the movement group of Barcelona en Comú is an online social community with an extreme ability to withstand or recover. At the same time the party group of Barcelona en Comú seems to only focus on the core users.

**Table 7:** Maximal and average *k*-index (standard deviation in parentheses) for the intra-network of each cluster in the network of retweets

Cluster	$k_\mathrm{max}$	$k_\mathrm{avg}$
${\rm BeC \text{-}m ^{\rm{rt}}}$	17	5.90 (5.46)
${\rm PP ^{\rm{rt}}}$	12	4.02 (3.99)
${\rm PSC ^{\rm{rt}}}$	11	3.85 (3.55)
${\rm CiU ^{\rm{rt}}}$	13	3.10 (3.44)
${\rm ERC ^{\rm{rt}}}$	8	2.25 (1.85)
${\rm Cs ^{\rm{rt}}}$	10	2.42 (2.42)
${\rm CUP ^{\rm{rt}}}$	10	2.19 (2.22)
${\rm BeC \text{-}p ^{\rm{rt}}}$	5	1.33 (0.71)

## Online party discussion networks

In this section we present the results of detecting and characterizing the major clusters in the network of replies, i.e., the online party discussion networks.

### Community detection

We apply the N-Louvain method on the network of replies. We should note that the network of retweets only contained edges with weight greater or equal to 3 while no threshold was applied for the network of replies. Given that the boundaries between online communities are fuzzier in this network, the method is applied by running the Louvain method 100 times and assigning to each cluster the nodes that fall into that cluster more than 50 times ($N=100, \varepsilon =0.5$), instead of 95 times as done for the network of retweets.

**Fig. 9:**
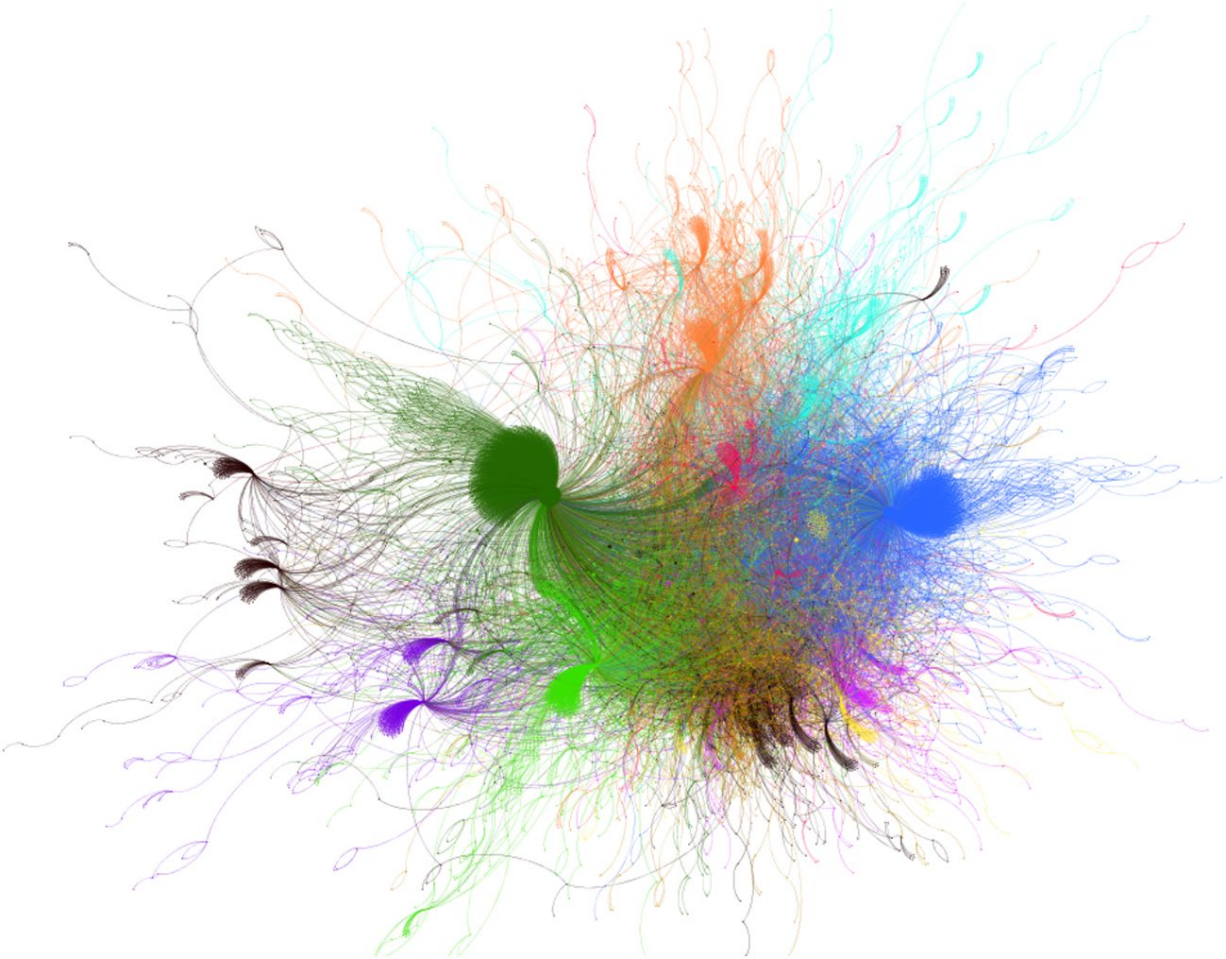
Network of replies distinguishing party clusters by color: ${\rm BeC \text{-}c ^{\rm{rp}}}$ (*dark green*); ${\rm BeC \text{-}p ^{\rm{rp}}}$ (*light green*); ${\rm Podemos ^{\rm{rp}}}$ (*purple*); ${\rm ERC ^{\rm{rp}}}$ (*yellow*); ${\rm PSC ^{\rm{rp}}}$ (*red*); ${\rm CUP ^{\rm{rp}}}$ (*violet*); ${\rm Cs ^{\rm{rp}}}$ (*orange*); ${\rm CiU ^{\rm{rp}}}$ (*dark blue*); ${\rm PP ^{\rm{rp}}}$ (*cyan*). *Brown* nodes belong to ${\rm Ind ^{\rm{rp}}}$, and *black* nodes belong to either ${\rm Media \text{-}Spa ^{\rm{rp}}}$ (*left*) or ${\rm Media \text{-}Cat ^{\rm{rp}}}$ (*bottom*)

**Fig. 10:**
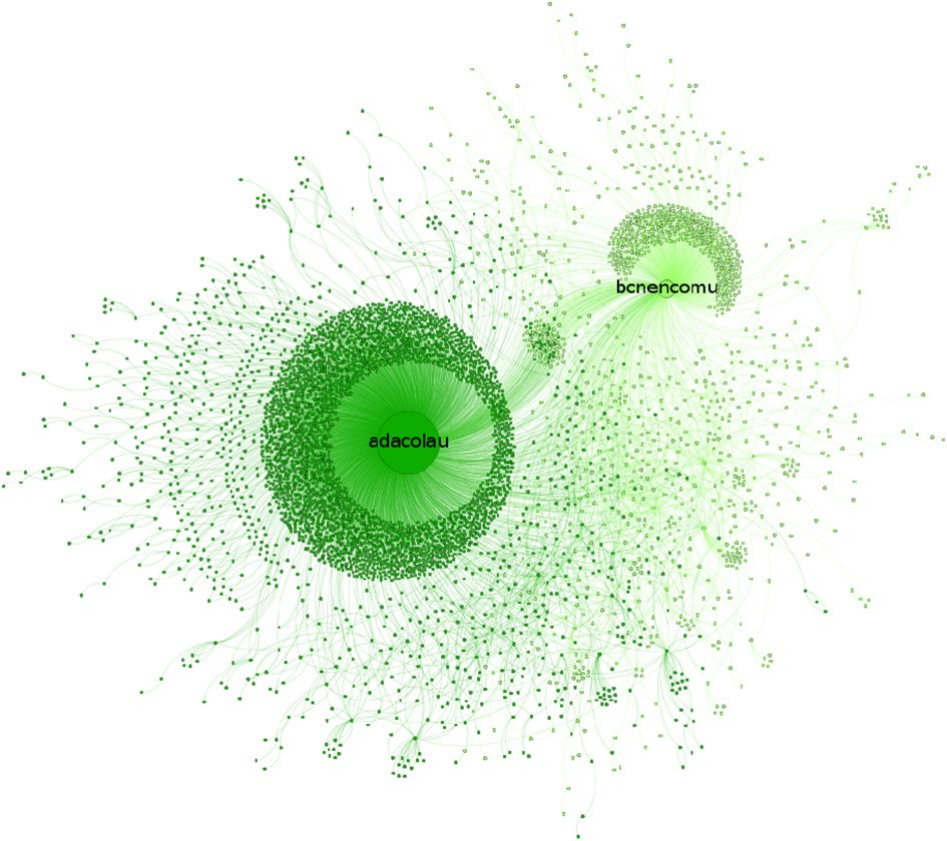
Sub-network of ${\rm BeC \text{-}c ^{\rm{rp}}}$ (*dark green*) and ${\rm BeC \text{-}p ^{\rm{rp}}}$ (*light green*). For better readability, the label of public users is shown

The network is presented in Fig. 9. For a better readability of the network, we only show the nodes that were assigned to a cluster with our method. By observing the most relevant node, according to PageRank, we first notice clusters around the leader of a party: ${\rm CiU ^{\rm{rp}}}$, ${\rm Cs ^{\rm{rp}}}$, ${\rm PSC ^{\rm{rp}}}$, ${\rm ERC ^{\rm{rp}}}$, ${\rm PP ^{\rm{rp}}}$, ${\rm CUP ^{\rm{rp}}}$, and ${\rm Podemos ^{\rm{rp}}}$ (member party of BeC). In the network of retweets Barcelona en Comú was divided in two clusters: movement and party. In the network of replies we also find two BeC clusters: one around the *candidate* account @adacolau (hereafter ${\rm BeC \text{-}c ^{\rm{rp}}}$), and another around the *party* account @bcnencomu (hereafter ${\rm BeC \text{-}c ^{\rm{rp}}}$). These two clusters are presented separately in Fig. 10.

**Fig. 11:**
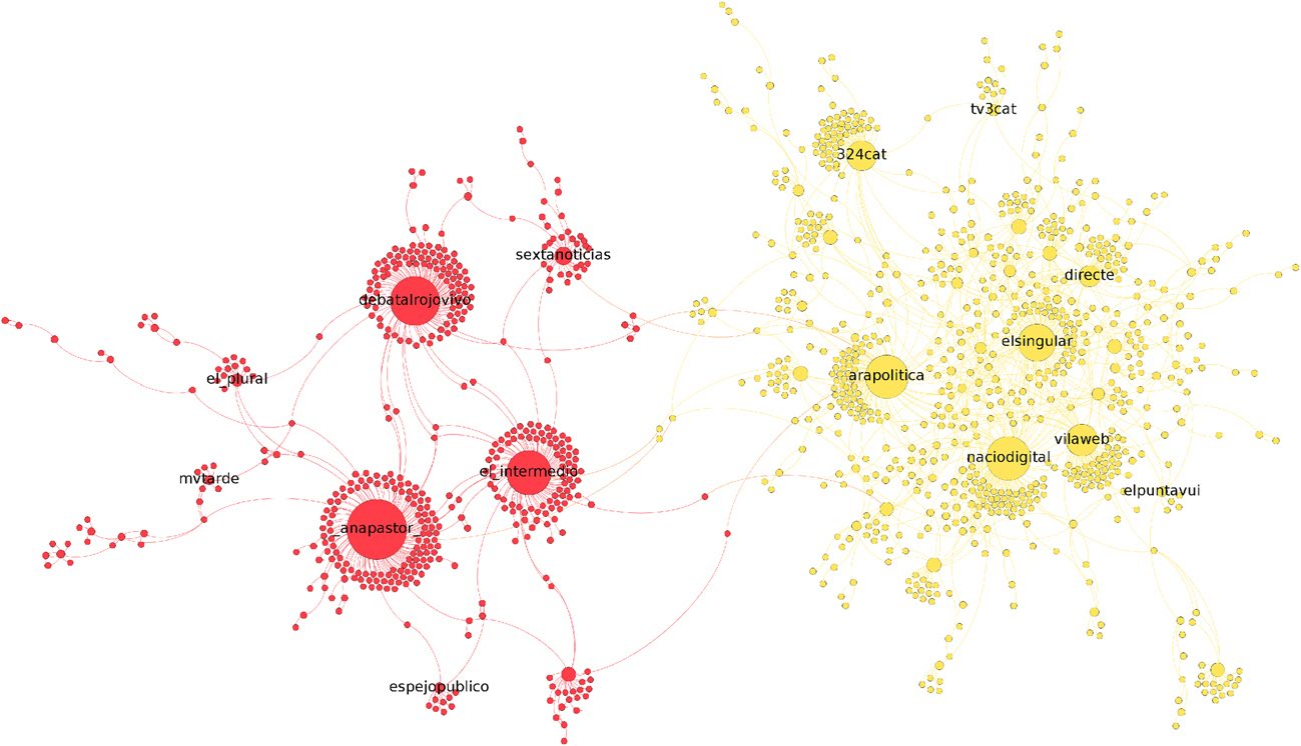
Sub-network of ${\rm Media \text{-}Spa ^{\rm{rp}}}$ (*red*) and ${\rm Media \text{-}Cat ^{\rm{rp}}}$ (*yellow*). For better readability, the label of public users is shown

In addition, the N-Louvain method ($N=100, \varepsilon =0.5$) in the network of replies detects other clusters which are worth examining. First, we obtain two clusters which, according to the nodes with highest PageRank, relate to media. This is different from the retweet network where we set $N=100$ and $\varepsilon =0.05$ to prevent the inclusion of media accounts in party clusters. We present the two media clusters using different colors in Fig. 11 to show that the main nodes in the red cluster are Spanish media, and the main nodes in the yellow cluster are Catalan media. For this reason, from now on, the analysis distinguishes these clusters as ${\rm Media{\text{-}}Spa ^{\rm{rp}}}$ and ${\rm Media{\text{-}}Cat ^{\rm{rp}}}$: Spanish media and Catalan media, respectively. We also observe that few interactions occur between the two clusters. Furthermore, our community detection method finds a large cluster, presented in Fig. 11, formed by users who advocate for the independence of Catalonia (hereafter ${\rm Ind ^{\rm{rp}}}$).

**Fig. 12:**
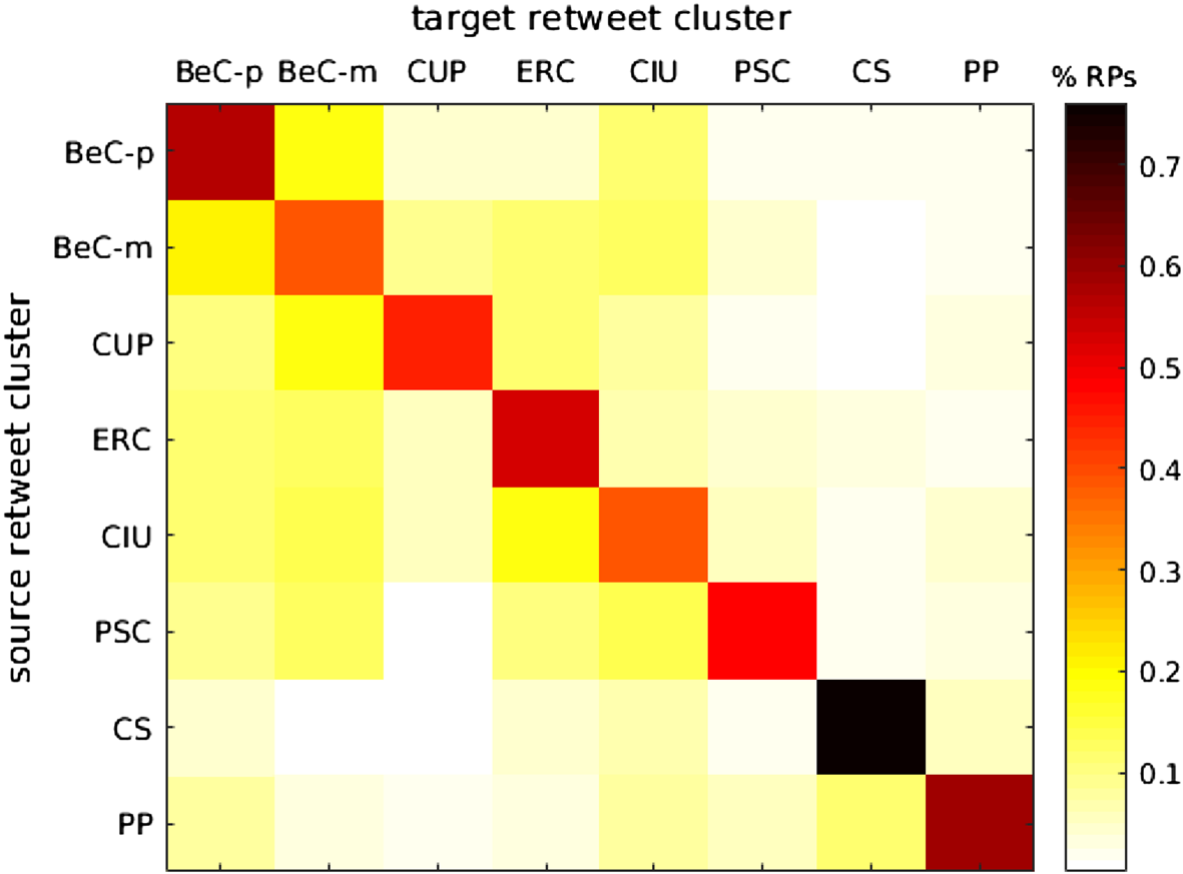
Normalized amount of replies between users from retweet clusters

#### Comparison to the network of retweets

We now compare replies and retweets between parties. First, we analyze the replies among the clusters of the retweet network. An interaction matrix *A* is presented in Fig. 12 where, now, an entry $A_{i,j}$ is the number of replies from users from cluster $i^\mathrm{rt}$ to users from cluster $j^\mathrm{rt}$. Although at first sight the vast majority of replies occurs in the main diagonal, like in Fig. 4, it is also evident that users are more likely to interact with users from other parties by replying than by retweeting them. Moreover, we observe behavioral differences between two types of parties. On the one hand, clusters of parties that advocate for a Catalan self-determination referendum (${\rm BeC \text{-}m ^{\rm{rt}}}$, ${\rm BeC \text{-}p ^{\rm{rt}}}$, ${\rm CiU ^{\rm{rt}}}$, ${\rm CUP ^{\rm{rt}}}$, ${\rm ERC ^{\rm{rt}}}$) exhibit a notable amount of inter-party replies. On the other hand, parties against the referendum (${\rm PSC ^{\rm {rt}}}$, ${\rm Cs ^{\rm{rt}}}$, ${\rm PP ^{\rm{rt}}}$) show a lower predisposition to dialogue with other parties and, therefore, most of their replies are within their own party.

Looking at each party individually, there are also observable differences. First, it can be seen that ${\rm BeC{\text{-}}m ^{\rm rt}}$ and ${\rm BeC{\text{-}p} ^{\rm rt}}$, especially the second, receive a higher amount of replies from the other parties than the rest. As previously noted, the parties in favor of a Catalan self-determination referendum interact more with each other; however, they exhibit different patterns: ${\rm CUP ^{\rm rt}}$, probably because it is a small party, generates more replies than it receives. It also interacts more with ${\rm BeC{\text{-}}m ^{\rm rt}}$, presumably due to their similar grassroots party nature. ${\rm ERC ^ {\rm {rt}}}$ gets larger attention from ${\rm CiU ^{\rm{rt}}}$ but the pattern is not symmetrical, as ${\rm CiU ^{\rm{rt}}}$ gets most of its attention from ${\rm BeC \text{-}m ^{\rm{rt}}}$ and ${\rm BeC \text{-}p ^{\rm{rt}}}$. It is also interesting to mention that ${\rm CiU ^{\rm{rt}}}$ is the cluster that has the highest proportion of inter-party interactions. ${\rm PSC ^{\rm{rt}}}$ follows a different pattern: on the one hand, its users reply to the two $\text{ BeC }$ clusters, ${ \rm CiU ^{\rm {rt}}}$ and ${\rm ERC ^{\rm{rt}}}$, neglecting ${\rm CUP ^{\rm{rt}}}$, (${\rm Cs ^{\rm{rt}}}$ and ${\rm PP ^{\rm{rt}}}$). On the other hand, it receives almost no replies from the other parties. Finally, the right wing parties (${\rm Cs ^{\rm{rt}}}$ and ${\rm PP ^{\rm{rt}}}$) appear as isolated political communities that do not interact with the other clusters: their proportion of intra-party interactions is the highest (especially Cs), and they write slightly more replies than they receive.

**Fig. 13:**
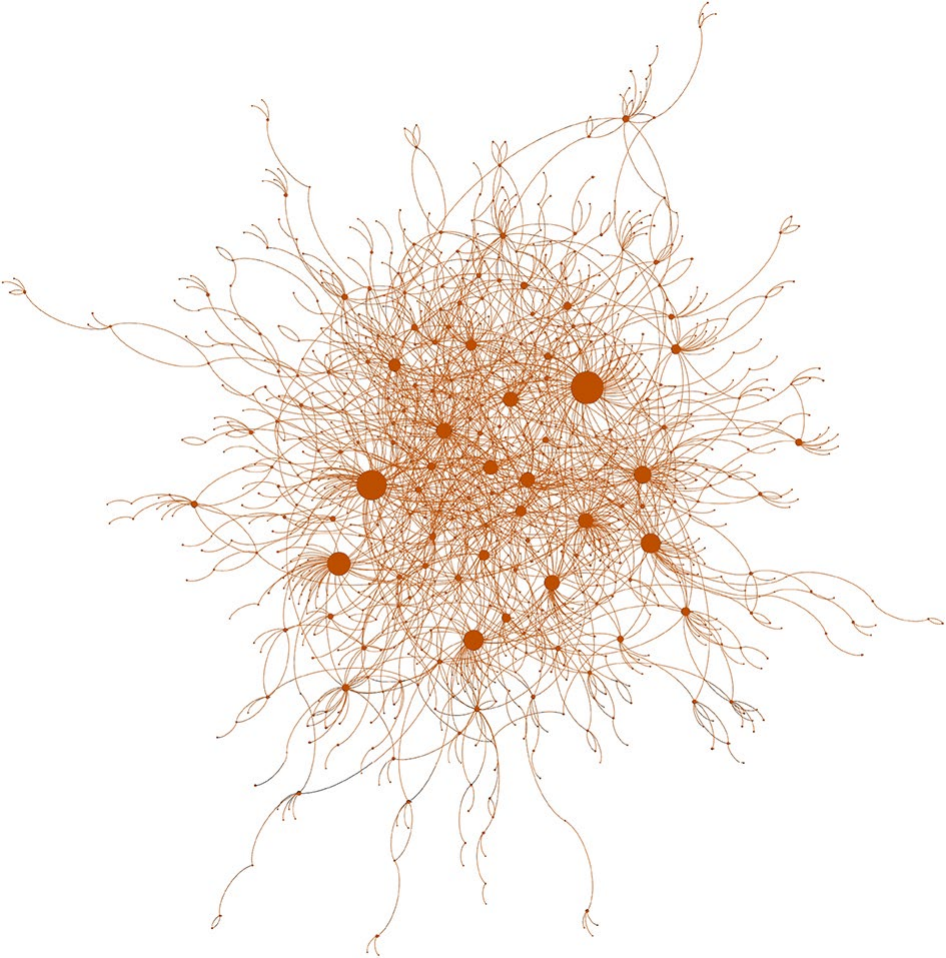
Sub-network of ${\rm Ind ^{\rm{rp}}}$

We then compare the clusters from the retweet network to the ones from the reply network. We present in Table 8 how many users from each reply cluster (rows) belong to each retweet cluster (columns); i.e., the party distribution of reply clusters. As expected, the majority of users were assigned to the corresponding cluster in the retweet network, consistent with the diagonal of the adjacency matrix in Fig. 12. It is important to point out the high amount of undefined users. This notorious difference is explained by multiple reasons. First, the retweet network only contains the interactions between any pair of users that occurred at least 3 times. Thus, the retweet network has only 6492 nodes, while the reply network has 21,846. Second, the sensibility level of the N-Louvain method is lower for the retweet network (0.05 vs 0.5), i.e., many users are undefined in the retweet network because they did not fall into the same cluster in more than 95% of the executions. Although the number of undefined users is high, users in retweet clusters might better represent party supporters. Therefore, this comparison becomes a good strategy to have a better understanding of the nature of replies between political parties.

We remark in Table 8 to which cluster of retweets (excluding undefined) the largest number of users of each reply cluster belong (bold values). This allows us to observe interesting patterns. In particular, we notice that most users from ${\rm BeC \text{-}c ^{\rm{rp}}}$ and ${\rm BeC \text{-}p ^{\rm{rp}}}$ are from Barcelona en Comú. When examining the table by columns, we see that users from ${\rm BeC \text{-}m ^{\rm{rt}}}$, ${\rm BeC \text{-}p ^{\rm{rt}}}$, ${\rm CUP ^{\rm{rt}}},$ and ${\rm ERC ^{\rm{rt}}}$ appear more frequently in other clusters. This leads us to consider that these users have a higher willingness to dialogue with users from other parties. This is observed particularly in the case of ${\rm BeC \text{-}p ^{\rm{rt}}}$, as its users occur in every reply cluster. However, when examining the table by rows, we see that ${\rm BeC \text{-}c ^{\rm{rp}}}$ and ${\rm CiU ^{\rm{rp}}}$ are the clusters with more diversity of users, which may indicate that they receive large attention from the others. This is coherent with the fact that the two parties represented the frontrunners in the election, and actually the outgoing and the forthcoming mayors.

Finally, we note that the two media clusters have completely different natures: ${\rm Media \text{-}Cat ^{\rm{rp}}}$ is mainly composed of users from parties advocating for a Catalan self-determination referendum: ${\rm ERC ^{\rm{rt}}}$, ${\rm CUP ^{\rm{rt}}}$, ${\rm CiU ^{\rm{rt}}},$ and both ${\rm BeC ^{\rm{rt}}}$. This is expected because ${\rm Media \text{-}Cat ^{\rm{rp}}}$ is formed around Catalan media outlets with higher sensitivity to Catalan political issues. Differently, the party that interacted most with ${\rm Media \text{-}Spa ^{\rm{rp}}}$ is BeC.

**Table 8:** Number of nodes from each cluster in the reply network (rows) which occur in each cluster in the retweet network (columns)

Cluster	${\rm BeC \text{-}m ^{\rm{rt}}}$	${\rm BeC \text{-}p ^{\rm{rt}}}$	${\rm CiU ^{\rm{rt}}}$	${\rm Cs ^{\rm{rt}}}$	${\rm CUP ^{\rm{rt}}}$	${ \rm ERC ^{\rm{rt}}}$	${\rm PP ^{\rm{rt}}}$	${\rm PSC ^{\rm{rt}}}$	Undef.
${ \rm BeC \text{-}c ^{\rm{rp}}}$	37	*140*	13	1	14	47	6	3	3259
${ \rm BeC \text{-}p ^{\rm{rp}}}$	104	*120*	4	0	24	13	0	4	937
${\rm CiU ^{\rm{rp}}}$	27	48	*108*	6	37	17	13	13	1975
${\rm Cs ^{\rm{rp}}}$	2	18	0	*100*	5	16	12	1	925
${\rm CUP ^{\rm{rp}}}$	7	6	0	1	*82*	7	1	1	314
${\rm ERC ^{\rm{rp}}}$	1	6	7	2	9	*113*	0	2	519
${\rm Ind ^{\rm{rp}}}$	14	18	18	1	16	*91*	0	0	807
${\rm Media \text{-}Cat ^{\rm{rp}}}$	2	12	6	0	10	*63*	1	4	669
${\rm Media \text{-}Spa ^{\rm{rp}}}$	0	*23*	0	1	1	1	1	0	432
${\rm Podemos ^{\rm{rp}}}$	0	*35*	1	0	0	0	1	1	440
${ \rm PP ^{\rm{rp}}}$	2	5	5	4	4	6	*80*	4	435
${\rm PSC ^{\rm{rp}}}$	2	4	6	1	5	13	1	*57*	396

The largest number of each row is in italics (undefined users are not considered)

### Cluster characterization

Finally, we characterize the clusters in the network of replies using the same metrics of the above section. The visualization of Fig. 10 exhibited the star-like structure of both clusters of Barcelona Comú, an effect that is accentuated in ${\rm BeC \text{-}c ^{\rm{rp}}}$. The results of the metrics present in Table 9 confirm that the Gini coefficient in ${\rm BeC \text{-}c ^{\rm{rp}}}$ ($G_\mathrm{in}$ = 0.980) is higher than in ${\rm BeC \text{-}p ^{\rm{rp}}}$ ($G_\mathrm{in}$ = 0.908). This might be produced by the attention to the candidate of Barcelona en Comú, who finally got elected as Mayor of Barcelona. Also, it is interesting to mention the structure of ${\rm Ind ^{\rm{rp}}}$, distinctive from the other clusters. One can observe its decentralized structure in Fig. 13. The metrics bear out this decentralized structure, having this cluster the lower in-degree inequality ($G_\mathrm{in}$ = 0.723), the largest clustering coefficient ($\rm Cl$ = 0.033), and the highest maximum and average *k*-index ($k_\mathrm{max}=5$, $k_\mathrm{avg}=1.58$). This might be an effect of not being a partisan cluster but one configured around a thematic political discussion. Finally, results also show that the structure of ${\rm Media \text{-}Cat ^{\rm{rp}}}$ ($G_\mathrm{in} = 0.899$, $\mathrm{Cl} = 0.008$, $k_\mathrm{max}=4$, $k_\mathrm{avg}=1.35$) is more decentralized than the structure of ${\rm Media \text{-}Spa ^{\rm{rp}}}$ ($G_\mathrm{in} = 0.974$, $\mathrm{Cl} = 0.001$, $k_\mathrm{max}=2$, $k_\mathrm{avg}=1.10$).

**Table 9:** Number of nodes (*N*) and edges (*E*), inequality based on the Gini coefficient ($G_\mathrm{in}$) of the in-degree distribution, clustering coefficient (Cl), average path length (*l*), maximal and average *k*-index (standard deviation in parentheses) for the intra-network of each cluster in the network of replies

Cluster	*N*	*E*	$G_\mathrm{in}$	Cl	*l*	$k_\mathrm{max}$	$k_\mathrm{avg}$
${\rm BeC \text{-}c ^{\rm{rp}}}$	3520	3940	0.980	0.0001	3.28	3	1.11 (0.36)
${\rm BeC \text{-}p ^{\rm{rp}}}$	1206	1624	0.908	0.0020	5.19	4	1.30 (0.64)
${\rm CiU ^{\rm{rp}}}$	2244	3446	0.849	0.0009	2.72	4	1.30 (0.60)
${\rm Cs ^{\rm{rp}}}$	1079	1478	0.900	0.0044	3.48	4	1.31 (0.67)
${\rm CUP ^{\rm{rp}}}$	419	528	0.865	0.0052	5.25	3	1.22 (0.49)
${\rm ERC ^{\rm{rp}}}$	659	841	0.927	0.0032	3.36	3	1.24 (0.51)
${\rm Ind ^{\rm{rp}}}$	965	1789	0.724	0.0333	5.37	5	1.58 (0.98)
${\rm Media \text{-}Cat ^{\rm{rp}}}$	767	1055	0.899	0.0076	3.57	4	1.35 (0.73)
${\rm Media \text{-}Spa ^{\rm{rp}}}$	459	499	0.974	0.0011	1.35	2	1.10 (0.31)
${\rm Podemos ^{\rm{rp}}}$	478	549	0.951	0.0013	1.79	2	1.12 (0.32)
${\rm PP ^{\rm{rp}}}$	545	709	0.876	0.0104	3.26	3	1.27 (0.55)
${\rm PSC ^{\rm{rp}}}$	485	614	0.892	0.0032	2.94	3	1.23 (0.49)

## Discussion

In this study we have proposed and validated a computational methodology to answer two research questions in relation to the Twitter party networks for the 2015 Barcelona City Council election. We discuss in this section the implications of our results.

### Institutionalization of a movement

The institutionalization of political parties is a research topic which has attracted much attention from scholars [8, 38, 39, 41, 53, 61]. The analysis of the network of retweets has been designed to answer the first research question that deals with the kind of organizational structure that Barcelona en Comú developed for the election campaign. On the one hand, the cited literature [30, 59] provided evidence of the decentralization of the 15M movement, which inspired the Barcelona en Comú candidacy. On the other hand, many political scientists [43, 44, 46, 51] argued that parties are historically ruled by elites and, therefore, result in centralized organizations. Furthermore, the historical models of political parties reviewed in [34] (i.e., *Caucus parties*, *Mass parties*, *Catch-all parties*, and *Cartel parties*) always assumed organization around elites. All of these observations motivated to study whether Barcelona en Comú preserved a decentralized structure, consistent with the decentralization of political power postulated in [14], or adopted a conventional centralized organization.

The results depict a movement-party structure in which the two components form well-defined clusters. In comparison to the clusters of the rest of political parties, the BeC movement community emerges as the least hierarchical, most clustered, and resilient one. In contrast, the BeC party community is the most hierarchical, least clustered, and least resilient one. The centralization of the party cluster points to the candidate and official accounts, the subjects that are commonly associated with the elite. However, unlike the rest of political parties, there is a co-existence of both party and movement clusters. This co-existence is consistent with the hypothesis expressed in [58] when defining Podemos, member party of Barcelona en Comú, as the conjugation of a front-end and a back-end.

This article has provided hints about the characterization of the organization of political parties according to their online diffusion networks. The nature of Barcelona en Comú is similar to the so-called niche parties because this party rejects the traditional class-based orientation of politics, does not fit with classical lines of political division, and is appealing to voters who may cross-cut classical partisan alignments [42]. Although niche parties are the result of institutionalized social movements (e.g., communism, green, nationalism) and differ from mainstream parties, Internet is not found relevant in their process of institutionalization [2]. In contrast, some authors have reported that the Internet played a key role in the organization of the 15M movement for building “a hybrid space between the Internet social networks and the occupied urban space” [13]. According to  [59], this hybrid space is the result of *techno-political* practices: “the tactical and strategic use of technological devices (including social networks) for organization, communication and collective action.” Are techno-politics the origin of this particular movement-party partition of Barcelona en Comú? Recently, political scientists have postulated the emergence of *cyber parties* “with its origins in developments in media and information and communication technologies” [40]. Although the results of this study cannot ensure that the Internet and social media are the only reason behind this new form of political organization, in this particular context some party activists reported that ICT becomes essential for campaigning [56]. Therefore, a close link between techno-politics and the structure of Barcelona en Comú might exist.

### Discussion behaviors

The analysis of the network of replies allows us to answer the second research question about the discussion behavior of Barcelona en Comú. Similar to the network of retweets, the results have depicted another dual structure: one around the candidate and another around the party account, which includes a large amount of 15M activists. Thus, while the candidate cluster received the larger attention from other parties, the party cluster presented a higher willingness to dialogue with other parties. Given that the rest of the parties are mainly organized in a single cluster, this dual structure confirms the different behavior of Barcelona en Comú when discussing with other political parties.

The results have also showed a non-partisan cluster with users associated with the Catalan independence movement. This is consistent with previous research that already indicated that online users do not have a strong preference to discuss with members of the same political party but to discuss around specific topics [23, 48]. In contrast, we have seen that the Spanish parties PP, PSC, and CS have a lower predisposition to dialogue with other parties. This result is of interest given that (1) the independence of Catalonia is a main topic of Spanish politics, and (2) the current Government of Spain is supported by these three parties. In addition, it has been observed that just a few users interacted with both Spanish and Catalan media. On the one hand, this could be an effect of the topics covered by the different types of media, e.g., Catalan issues are expected to be more frequent in Catalan media outlets. On the other hand, this could be also an idiomatic issue, i.e., Catalan native speakers are more likely to interact with media outlets tweeting in Catalan. In general, our analysis depicts the existence of groups of opposing views. Therefore, this scenario could be used to evaluate recent approaches for balancing opposing views to reduce controversy in social media [26].

### Contribution of our methodology

The methodology of this article focuses on (1) community detection and (2) cluster characterization. The fuzzy membership of some nodes in certain communities (e.g., media accounts in the party clusters from the retweet network) motivated the modification of a standard community detection algorithm (Louvain method) by setting a sensibility level to parametrize the robustness of the final clusters. In comparison to the standard Louvain method and another community detection algorithm for overlapping communities (Clique Percolation Method), the evaluation proved that the new algorithm identified the political networks in a more stable way. Cluster characterization was inspired by the metrics proposed in [23] to compare political party networks. The original dimensions of this framework were hierarchical structure, information efficiency, and social resilience. The redefinition of these three dimensions and the inclusion of new metrics constitute an improvement of the characterization of political networks:

- *Hierarchical structure* In-degree centralization [22] was originally applied in [23] to measure the hierarchical structure of a network. This metric is based on (1) how the centrality of the most central node exceeds the centrality of all other nodes and (2) the comparison to a star network. Maximum and average in-degree have common differences of several orders of magnitude in social graphs. Therefore, in-degree centralization is approximately equal to the ratio between the maximum in-degree and the number of nodes for social networks with a heavy-tailed in-degree distribution. In other words, the in-degree centralization is not a good metric to capture hierarchical structure for social diffusion graphs, and the Gini coefficient for in-degree inequality represents a more reliable measure of the hierarchical structure of a network.
- *Information efficiency* Information efficiency in social networks is closely related to the *small-world phenomenon*. This article uses the average path length, as the previous framework does [23], and the clustering coefficient to better characterize efficiency in social networks.
- *Social resilience* Previous studies indicated the suitability of the *k*-core decomposition to measure the resilience of social networks [24]. This framework recommends the term *coreness* which represents a more precise definition of this metric. In addition, showing the distribution of nodes along *k*-cores does capture resilience better than maximum *k*-core as done in [23].

## Conclusion

In this article we have examined new forms of political organization in social media. The results focus on the Twitter networks of Barcelona en Comú in comparison to the other parties for the 2015 Barcelona municipal elections. The findings rely on a dataset from Twitter but social networks are only a slice of the structure of political organizations and not every party activist has a Twitter account. Furthermore, some experts are skeptical with the digital forms of activism because of the “loss of coherence, morality or even sustainability” [45] and pointed out the rise of a low commitment and feel-good form of activism. Nevertheless, online platforms are playing a key role in political discussion and campaigning, and social media data are leveraging the capacity of revealing patterns of individual and group behaviors [29, 35]. Because of the Internet’s potential for increasing debate in political parties [60] and the potential relevance of low commitment online participants for collective action [6], Twitter might be seen as an informative and valuable data source to examine collective behavior and self-organization in social and political contexts.

The results showed that the tension between the decentralization of networked movements and the centralization of political parties led to a movement-party structure: both paradigms co-exist in two well-defined clusters. From this result, future work should investigate the origin of this particular structure by adding longitudinal analyses of the formation of the clusters. Furthermore, it is interesting to note that city council elections were held in every Spanish city in May 2015 and candidacies similar to Barcelona en Comú were built. Indeed, similar organizations (e.g., Ahora Madrid, Zaragoza en Común) obtained the Government of many of the largest Spanish cities. For this reason and because relevant studies on political parties in advanced industrial democracies often ignore the Spanish context [4, 17], future work might apply this framework to examine whether the characteristics observed in Barcelona en Comú are also present in these other Spanish grassroots movement-parties.

## Abbreviations

API: Application Programming Interface
BeC: Barcelona en Comú
CiU: Convergència i Unió
Cs: Ciudadanos
CUP: Capgirem Barcelona
ERC: Esquerra Republicana de Catalunya
PP: Partit Popular de Catalunya
PSC: Partit dels Socialistes de Catalunya

## Footnotes

1
https://barcelonaencomu.cat/.

2
http://www.ciu.cat/.

3
https://www.ciudadanos-cs.org/.

4
http://cup.cat/.

5
http://www.esquerra.cat/.

6
http://www.ppcatalunya.com/.

7
http://www.socialistes.cat/.

8
http://www.cfinder.org/.
